# Perioperative Use of Levosimendan in Hepatic Transplantation With Diastolic Heart Failure: A Case Report

**DOI:** 10.7759/cureus.59490

**Published:** 2024-05-01

**Authors:** Erick Villafán Vázquez, Ricardo Acuña Razo, Mayra Michelle Nuñez Rueda

**Affiliations:** 1 Anesthesiology, University Faculty of Medicine Mexicana La Salle, Mexico City, MEX; 2 Anesthesiology, Unidad Médica de Alta Especialidad (UMAE) Hospital de Especialidades Centro Médico Nacional "La Raza", Mexico City, MEX; 3 Anesthesiology, XXI Century National Medical Center, Mexican Institute of Social Security (IMSS), Mexico City, MEX; 4 Neuroanesthesiology, National Institute of Neurology and Neurosurgery, Mexico City, MEX

**Keywords:** perioperative anesthesia, anesthesia, heart failure, transplant, levosimendan

## Abstract

Levosimendan is a medication with a range of pharmacological effects, making it appropriate for use in several clinical settings, including advanced heart failure with pulmonary hypertension, cardiogenic shock, and acute heart failure. This case report details the perioperative management of a male in their 40s with a complex medical history, including primary hypoparathyroidism, cirrhosis, and severe pulmonary hypertension, who underwent urgent cadaveric donor liver transplantation. Information available on the perioperative use of levosimendan is still insufficient to be able to regulate behaviors that can guide its management on a regular basis.

## Introduction

Known for its beneficial inotropic effects on the heart and vasodilation in the vascular system, levosimendan is a class III derivative [1,2]. Its inotropic activity is related to the calcium sensitization of contractile proteins, which increases the stability of troponin C-Ca2+ complexes and increases the synthesis of actin and myosin cross-bridges [1,2]. Levosimendan has a short elimination half-life of around one hour, but because its active metabolite OR-1896 has an approximately 80-hour half-life, its hemodynamic effects can last for many hours [3,4].

It has a threefold mechanism of action that makes it an inodilator that promotes positive inotropy while preserving neutral oxygen consumption. Additionally, it has properties that include preconditioning, cardioprotective, anti-stunned, and anti-ischemic [4]. The effects on cardiac surgery patients with and without prior systolic dysfunction were assessed in a recent meta-analysis by Harrison and colleagues [5]. The study found that levosimendan therapy decreased mortality and other negative outcomes, such as postoperative atrial fibrillation, myocardial damage, and postoperative renal failure requiring dialysis. Patients having a lower left ventricular ejection fraction (LVEF) saw these advantages the greatest [6]. 

There are short-term and long-term impacts of cardioprotection. Preconditioning, postconditioning, anti-stun, and anti-ischemic actions all contribute to short-term cardioprotection. Effects that are anti-remodeling, anti-apoptotic, and anti-inflammatory contribute to long-term cardioprotection. Levosimendan preconditioning causes mitochondrial KATP channels to open, lowering calcium buildup and stabilizing mitochondrial inner membrane permeability. This prevents ischemia and reperfusion harm to the myocardium. In animal models, levosimendan preconditioning has been demonstrated to drastically decrease infarct size and increase survival rates. Levosimendan has anti-stun effects in patients with acute coronary syndrome and myocardial [5]. 

The elimination half-life of levosimendan is only around an hour. It takes around four hours of continuous infusion to attain a steady-state concentration without a loading dose. Levosimendan's serum levels drop rapidly when the infusion is finished. Levosimendan does, however, have an active circulating metabolite known as OR-1896, which has an approximately 80-hour half-life for elimination. According to research by Lilleberg and colleagues, a 24-hour levosimendan medication enhanced cardiac output and decreased pulmonary capillary wedge pressure in patients with heart failure [3,5-8]. 

In the context of hepatic transplantation, administering levosimendan during the reperfusion stage has been shown to boost the metabolic function of human liver cells and lower AST enzyme levels, indicating reduced liver damage. Furthermore, cells treated with levosimendan exhibit decreased apoptosis rates following ischemia-reperfusion injury (IRI) compared to untreated cells. This protective effect is partly attributed to levosimendan's ability to maintain levels of the anti-apoptotic protein Bcl-2 and prevent the increase of the pro-apoptotic protein BAX, enhancing cell survival post-transplantation [9].

## Case presentation

A male in their 40s with a diagnosis of alcoholic cirrhosis diagnosed in 2019, Model for End-Stage Liver Disease-sodium (MELD-NA) score of 11 points, Duke Activity Status Index (DASI) score of 9.95 points with 3.97 METS and VO2 max 17 ml/kg/min, New York Heart Association (NYHA) classification of III, Barthel score of 100 points, and Pfeiffer test of 0 points, currently undergoing treatment with spironolactone 100 mg/24 hours, furosemide 40 mg/12 hours, and propranolol 15 mg/12 hours, was urgently admitted for a cadaveric donor liver transplantation. He has a history of primary hypoparathyroidism diagnosed in 1998 and is being treated with levothyroxine 100 mcg/24 hours Mondays to Saturdays and 50 mcg/24 hours Sundays, currently under control.

An echocardiogram performed on April 26, 2023, reported a dilated left ventricle with a left ventricular end-diastolic diameter of 58 millimeters and a left ventricular end-systolic diameter of 36 millimeters. The velocity of the E wave was 1.35 meters per second, the velocity of the A wave was 0.59 meters per second, and the E-to-A ratio was 2.2. The patient had preserved systolic function and type III diastolic dysfunction. Elevated filling pressures were noted, along with dilated right heart chambers. There were severe left atrial dilation by volume, mild mitral and tricuspid regurgitation, and mild pulmonary regurgitation with an estimated systolic pulmonary artery pressure by echocardiography of 51 mmHg, suggesting moderate pulmonary hypertension. Right ventricular systolic function was preserved as indicated by Tricuspid Annular Plane Systolic Excursion (TAPSE).

Upon admission to the operating room, monitoring was initiated, including noninvasive blood pressure (BP), pulse oximetry, 5-lead electrocardiogram, SedLine neuromonitoring, and cerebral oximetry monitoring with near-infrared spectroscopy (NIRS). Vital signs reported were as follows: heart rate (HR) 50 bpm, BP 90/58 mmHg, and SPO2 97%. 

Anesthesia induction was achieved with fentanyl 4 mcg/kg, propofol 1 mg/kg, cisatracurium 0.1 mg/kg, and lidocaine 1 mg/kg. Intubation was performed without complications using a video laryngoscope.

Subsequently, a right internal jugular central venous catheter, Swan-Ganz floating catheter, right radial arterial line with FloTrac sensor, and HemoSphere platform monitor were placed. The patient's cardiac output was 3 L/min, cardiac index 1.4 L/min/m², pulmonary artery pressure 22/19 mmHg, pulmonary capillary wedge pressure 20 mmHg, systemic vascular resistance 1963 dyn·s/cm⁵, mixed venous oxygen saturation 66%, mean arterial pressure 63 mmHg, pleth variability index 13%, stroke volume variation less than 10%, and central venous pressure 28 mmHg.

Transesophageal echocardiography was not feasible due to esophageal varices and recent bleeding history.

Anesthesia was maintained with sevoflurane titrated to 0.7 minimum alveolar concentration guided by a processed electroencephalogram. During the intraoperative period, fentanyl was infused at 0.039 mcg/kg/min, lidocaine 20 mcg/kg/min, and dexmedetomidine 0.1 mcg/kg/hr. Cisatracurium was administered at 7.14 mcg/kg/min.

Fluid therapy was guided by dynamic goals and included PlasmaLyte, 5% glucose solution, sodium chloride 0.9%, albumin, and blood products.

Hemodynamic support during the hepatectomy phase consisted of dobutamine 5 mcg/kg/min, norepinephrine 0.3 mcg/kg/min, and vasopressin 0.08 UI/kg/min. These agents were titrated based on dynamic variables and blood gas analysis to maintain adequate perfusion.

Before the administration of levosimendan infusion, the patient's parameters were as follows: heart rate 51 bpm, blood pressure 81/58 mmHg, oxygen saturation 97%, pleth variability index 9, stroke volume variation less than 10%, central venous pressure 30 mmHg, cardiac index 1.8 L/min/m², mean arterial pressure 60 mmHg, cardiac output 2.8 L/min, cardiac index 1.4 L/min/m², pulmonary artery pressure 22/19 mmHg, pulmonary capillary wedge pressure 20 mmHg, systemic vascular resistance 1963 dyn·s/cm⁵, venous oxygen saturation 66%, mean arterial pressure 63 mmHg, pleth variability index 13, stroke volume variation less than 10, and central venous pressure 28 mmHg. The infusion which started at 0.2 mcg/kg/min was added electively prior to portal-caval clamping to reduce vascular resistance during the anhepatic phase to prevent decompensation of right heart failure in this patient, maintaining dynamic and static parameters but reducing the requirement of inotropes.

Just before unclamping, mean arterial pressure was maintained at 80 mmHg, central venous pressure 25 mmHg, pulmonary artery occlusion pressure 35 mmHg, and cardiac index 5 L/min/m². Following unclamping, mean arterial pressure decreased to 70 mmHg, central venous pressure to 16 mmHg, pulmonary artery occlusion pressure to 27 mmHg, and cardiac index to 2.9 L/min/m². Hemodynamic stability was maintained during the neohepatic phase.

The surgical procedure was successfully completed, and the patient was transferred to the intensive care unit without hemodynamic support where extubation was successfully performed on the fifth postoperative day.

**Figure 1:**
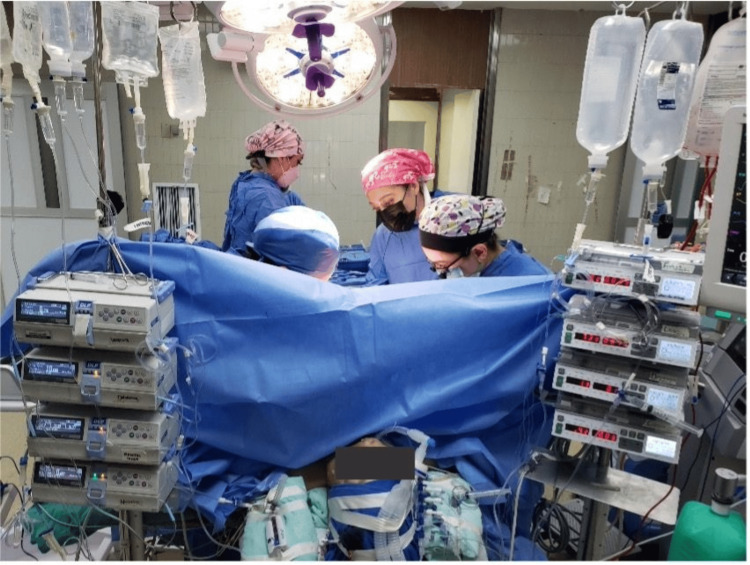
Anesthesia management for hepatic transplantation

Figure 1 shows the anesthesia management for hepatic transplantation.

## Discussion

The significance of elevated cardiac filling pressures in patients scheduled for liver transplantation encompasses several crucial facets that demand meticulous attention: Before the procedure, detecting heightened cardiac filling pressures can signal underlying cardiovascular issues like heart failure or pulmonary hypertension, influencing the patient's suitability for transplantation. Identifying these conditions beforehand allows for proper management and optimization of cardiovascular health prior to surgery. During the transplantation process, maintaining adequate cardiac filling pressures becomes paramount to ensure optimal organ perfusion and oxygen delivery. Elevated filling pressures may suggest insufficient cardiac output or other hemodynamic challenges, necessitating swift intervention to prevent perioperative complications. Following transplantation, patients face potential complications such as fluid overload, renal impairment, and cardiovascular instability. Monitoring cardiac filling pressures postoperatively aids in assessing fluid status, guiding fluid management strategies, and detecting early signs of cardiac or fluid-related issues requiring intervention. Furthermore, elevated cardiac filling pressures can impact graft function by affecting hepatic perfusion and oxygenation. Inadequate cardiac output might compromise blood flow to the transplanted liver, potentially leading to ischemia and graft dysfunction. Hence, vigilant monitoring of these pressures is vital for optimizing graft function and ensuring favorable transplant outcomes. Lastly, heightened cardiac filling pressures may increase the risk of postoperative complications like hepatic artery thrombosis, acute kidney injury, and pulmonary edema. Close monitoring and timely intervention are crucial to mitigate these risks and enhance patient outcomes throughout the perioperative period.

The use of levosimendan in this case was intended to harness the effects produced by the vasodilation mechanism, as in liver transplantation, before unclamping, it is necessary to bring the patient to supraphysiological hemodynamic levels. In patients with diastolic insufficiency, this supraphysiological state can further deteriorate cardiac function just before hepatic reperfusion. By increasing vascular capacitance, both volume and pressure cardiac overload could be avoided. Levosimendan increases metabolic activity and attenuates hepatocyte apoptosis. As a secondary outcome, the IRI is a frequent cause of hepatic failure after transplantation. Within the early stages of reperfusion, microcirculation failure is brought about by endothelial cell swelling [8] and vasoconstriction. Upregulation of endothelin-1 and downregulation of nitric oxide (NO) lead to vasoconstriction of the sinusoidal lumen. Brunner and colleagues reported that treatment with levosimendan under normoxic conditions reduced apoptosis in human hepatocytes and that this effect was particularly pronounced at high levosimendan dosages as proven by the fact that the greatest reduction of apoptosis was determined at all points in time in those cells treated with high levosimendan concentrations. In cell populations exposed to IRI, levosimendan was additionally found to reduce the total number of apoptotic cells in comparison with populations not exposed to levosimendan [9]. The innovative use of levosimendan in this setting, aimed at increasing vascular capacitance to manage the hemodynamic changes associated with liver transplantation, particularly during the anhepatic phase, underscores the importance of tailored pharmacological strategies in transplant anesthesia. Levosimendan's role in reducing hepatocyte apoptosis and potentially mitigating IRI further highlights its utility in liver transplantation, offering a dual benefit of managing cardiac function and supporting liver recovery. This case emphasizes the significance of a multidisciplinary approach in managing patients with multiple comorbidities undergoing major surgeries like liver transplantation. The successful outcome, characterized by the patient's stable postoperative recovery and extubation on the fifth day, reflects the efficacy of the anesthetic and surgical strategies employed, including meticulous hemodynamic monitoring and judicious use of pharmacological agents. Future research and clinical experiences with levosimendan in liver transplantation could provide deeper insights into its therapeutic potential, optimizing patient outcomes in similar high-risk surgical procedures.

## Conclusions

The case report highlights the use of levosimendan in a liver transplant patient. There are potential benefits for cardiac function and liver recovery; however, it's important to note that comprehensive evidence on levosimendan's perioperative use in such settings is limited. This underscores the need for further research to establish clear guidelines for its application in liver transplantation and similar complex surgeries, ensuring safe and effective patient care.
